# The Grape *VlWRKY3* Gene Promotes Abiotic and Biotic Stress Tolerance in Transgenic *Arabidopsis thaliana*

**DOI:** 10.3389/fpls.2018.00545

**Published:** 2018-04-25

**Authors:** Rongrong Guo, Hengbo Qiao, Jiao Zhao, Xianhang Wang, Mingxing Tu, Chunlei Guo, Ran Wan, Zhi Li, Xiping Wang

**Affiliations:** ^1^State Key Laboratory of Crop Stress Biology in Arid Areas, College of Horticulture, Northwest A&F University, Xianyang, China; ^2^Key Laboratory of Horticultural Plant Biology and Germplasm Innovation in Northwest China, Ministry of Agriculture, Northwest A&F University, Xianyang, China; ^3^Guangxi Academy of Agricultural Sciences, Nanning, China

**Keywords:** abiotic stress, biotic stress, grape, transgenic, *VlWRKY3*

## Abstract

WRKY transcription factors are known to play important roles in plant responses to various abiotic and biotic stresses. The grape WRKY gene, *WRKY3* was previously reported to respond to salt and drought stress, as well as methyl jasmonate and ethylene treatments in *Vitis labrusca* × *V. vinifera* cv. ‘Kyoho.’ In the current study, WRKY3 from the ‘Kyoho’ grape cultivar was constitutively expressed in *Arabidopsis thaliana* under control of the cauliflower mosaic virus 35S promoter. The *35S::VlWRKY3* transgenic *A. thaliana* plants showed improved salt and drought stress tolerance during the germination, seedling and the mature plant stages. Various physiological traits related to abiotic stress responses were evaluated to gain further insight into the role of *VlWRKY3*, and it was found that abiotic stress caused less damage to the transgenic seedlings than to the wild-type (WT) plants. *VlWRKY3* over-expression also resulted in altered expression levels of abiotic stress-responsive genes. Moreover, the *35S::VlWRKY3* transgenic *A. thaliana* lines showed improved resistance to *Golovinomyces cichoracearum*, but increased susceptibility to *Botrytis cinerea*, compared with the WT plants. Collectively, these results indicate that *VlWRKY3* plays important roles in responses to both abiotic and biotic stress, and modification of its expression may represent a strategy to enhance stress tolerance in crops.

## Introduction

Plants are regularly exposed to a broad range of abiotic and biotic stresses, such as drought, high salinity, extreme temperatures, and pathogen infection (Mishra et al., 2015). These stresses, which can occur concurrently, affect growth and development, can also alter species distribution (Shi et al., 2014). Plants have evolved diverse adaptive mechanisms to avoid or tolerate stress (Schmidt et al., 2010), including the recognition of stress cues and the transduction of the signals that regulate the expression of stress-related genes (Chinnusamy et al., 2004). Understanding the molecular basis of tolerance associated with multiple stresses, and identifying candidate genes that confer tolerance without compromising yield are goals in the development of crop varieties with enhanced tolerance of deleterious environmental factors (Atkinson and Urwin, 2012).

WRKY transcription factors comprise a large family of regulatory proteins that have been shown to play important roles in responses to biotic and abiotic stresses (Chen et al., 2009; Wang C. et al., 2013). A defining characteristic of WRKY transcription factors is s 60 amino acids WRKY domain, which is composed of a highly conserved WRKYGQK motif at the N-terminus, as well as zinc-finger-like motifs (either CX_4-5_CX_22-23_HXH or CX_7_CX_23_HXC) at the C-terminus (Eulgem et al., 2000; Rushton et al., 2012). The conserved WRKY domain is essential for recognizing and binding to a DNA *cis*-element, named the W-box, in the promoter regions of target genes (Sun et al., 2003).

Previous loss- and gain-of-function studies have demonstrated that WRKY transcription factors act as either positive or negative regulators in response to abiotic or/and biotic stresses (Eulgem et al., 2000; Pandey and Somssich, 2009; Rushton et al., 2010; Chen et al., 2012). Examples of biotic stress induced WRKY genes from *Arabidopsis thaliana* includes *WRKY3* and *WRKY4*, which promote resistance to necrotrophic pathogens, while *WRKY4* has been shown to suppress resistance to biotrophic pathogens (Lai et al., 2008). *WRKY8* has been reported to decrease resistance to the bacterial pathogen *Pseudomonas syringae*, but to promote resistance to the necrotrophic fungus *Botrytis cinerea* (Chen L.G. et al., 2010). In addition, *WRKY11* and *WRKY17* also suppress basal resistance to *P. syringae* in *A. thaliana* (Journot-Catalino et al., 2006), while constitutive expression of *WRKY18* enhances resistance to this pathogen, except when it is coexpressed with *WRKY40* or *WRKY60*, which results in the transgenic plants being more susceptible to both *P. syringae* and *B. cinerea* (Xu et al., 2006). *WRKY18, WRKY40* and *WRKY60* have also been reported to be involved in responses to ABA and abiotic stress by acting as either transcription activator or repressor (Chen H. et al., 2010). *WRKY57* and *WRKY63* were shown to have important roles in responses to drought stress (Ren et al., 2010; Jiang et al., 2012). A co-operative interaction between the *WRKY70* and *WRKY54* genes has also been identified in suppression of osmotic stress tolerance (Li et al., 2012). Finally, it has been suggested that *WRKY30* plays a role in responses to several abiotic or biotic stresses (Scarpeci et al., 2013).

WRKY genes from a range of crop species have also been implicated in responses to biotic and abiotic stresses including examples from rice (*Oryza sativa*, (Liu et al., 2005, 2007; Shimono et al., 2007; Qiu et al., 2007; Peng et al., 2008, 2012; Zhang et al., 2008; Abbruscato et al., 2012; Chujo et al., 2013; Wang H.H. et al., 2013; Yokokani et al., 2013), wheat (Wang C. et al., 2013), tomato (Bhattarai et al., 2010; Atamian et al., 2012; Liu et al., 2014), and cotton (Yu et al., 2012; Shi et al., 2014). We have been studying WRKY genes from grape (*Vitis vinifera* L.), one of the most economically important fruit crops in many countries (Wang et al., 2014), and in previous study, we identified 59 WRKY genes in the grape genome have been identified (Guo et al., 2014), however, only a few of these have been functionally characterized to date. *VvWRKY1* was reported to be involved in defense against downy mildew (Marchive et al., 2007, 2013), while over-expression of *VvWRKY2* in tobacco (*Nicotiana tabacum* cv. Xanthi) enhanced broad resistance to necrotrophic fungal pathogens. Moreover, the over-expression of *VvWRKY2* exhibited altered expression of genes involved in lignin biosynthesis pathway and cell wall formation, suggesting a role of *VvWRKY2* in the responses to biotic and abiotic stresses (Mzid et al., 2007; Guillaumie et al., 2010). In another study, the ectopic expression of two WRKY genes from *Erysiphe necator*-resistant Chinese wild *V. pseudoreticulata* W. T. Wang ‘Baihe-35-1,’ *VpWRKY1* and *VpWRKY2* in *A. thaliana* enhanced resistance to powdery mildew, and increased salt tolerance and/or cold tolerance (Li et al., 2010b). Another WRKY gene, *VpWRKY3* (corresponding to *WRKY10* in our previous study (Guo et al., 2014), also isolated from *V. pseudoreticulata* ‘Baihe-35-1,’ was also shown to participated in abiotic and biotic stress responses (Zhu et al., 2012). In addition, over-expression of *VvWRKY11* in *A. thaliana* enhanced dehydration stress tolerance (Liu et al., 2011), and *VvWRKY33* was reported to be involved in defense against the oomycete pathogen *Plasmopara viticola* (Merz et al., 2015).

We previously found that the expression of *WRKY3*, encoding a putative Group IIc WRKY gene, was up-regulated in *V. labrusca* × *V. vinifera* cv. ‘Kyoho’ following salt and drought stress treatments, as well as exogenous application of MeJA and Eth (Guo et al., 2014). In the current study, we examined the putative roles of *WRKY3* from ‘Kyoho’ grape in resistance to abiotic and biotic stresses by over-expressing the gene in *A. thaliana* and characterizing the responses of the resulting transgenic plants to drought and salt stresses, as well as to inoculation with *Golovinomyces cichoracearum*, and *B. cinerea*.

## Materials and Methods

### Plant Materials and Pathogen Bacteria

Two year old *V. labrusca* × *V. vinifera* cv. ‘Kyoho’ seedlings were grown in a greenhouse at the Northwest A&F University, Yangling, Shaanxi, China, and used as a source of material to clone *WRKY3*. *A. thaliana* L. ecotype Col-0 was used for the over-expression experiments and the WT and transgenic *A. thaliana* lines were grown at 22°C, 70% relative humidity and a long-day photoperiod (16 h light/8 h dark).

*Arabidopsis thaliana* powdery mildew (*G. cichoracearum* isolate UCSC1) was cultured on the *A. thaliana pad4* mutant plants at 22°C, and a photoperiod of 16 h light/8 h dark. *B. cinerea* was maintained at 22°C on Potato Glucose Agar as described by Wan et al. (2015).

### *VlWRKY3* Cloning and Sequence Analysis

Total RNA was extracted from the leaves of ‘Kyoho’ using the E.Z.N.A. Plant RNA Kit (Omega Bio-tek, United States, R6827-01), following the manufacturer’s protocol. First-strand cDNA was synthesized using PrimeScript^TM^ RTase (TaKaRa Biotechnology, Dalian, China) according to the manufacturer’s instructions. A *VlWRKY3* cDNA (GeneBank accession number: XM_002275540.3) corresponding to the predicted ORF was amplified by PCR using the gene-specific primers F1 (5′- ATG GAT AGC TTC TCC ACT C-3′) and R1 (5′-TTA AAA GGA AGC ATA AAC TTG C-3′). The PCR product was cloned into the pGEM^®^ -T Easy vector (Promega, Madison, WI, United States), and the recombinant plasmid was sequenced and named pGEM^®^-T Easy-*VlWRKY3*. Amino acid sequences of homologous WRKY3 proteins from other plant species were obtained from the NCBI database^1^ using BLASTP. A multiple sequence alignment of the deduced protein sequences and phylogenetic analyses were carried out using the DNAMAN software. Domain prediction and the logo representation of the WRKY domain was performed using MEME^2^.

### Generation of Transgenic *A. thaliana* Plants Over-Expressing the Grapevine *WRKY3* Gene

The coding sequence of *VlWRKY3* (with *Xba*I and *Kpn*I sites added to its 5′ and 3′ ends, respectively) was amplified from pGEM-Teasy-*VlWRKY3* using gene-specific primers F2 (5′-GCTCTAGA ATGGATAGCTTCTCCACTC-3′; *Xba*I site underlined) and R2 (5′-GGGGTACC TTAAAAGGAAGCATAAACTTGC-3′; *Kpn*I site underlined), and inserted immediately downstream of the CaMV 35S promoter in the plant over-expression vector, pCambia 2300 (Cambia, Brisbane, QLD, Australia), termed as *35S::VlWRKY3*. *Agrobacterium tumefaciens* GV3101 harboring *35S::VlWRKY3* constructs was used to transform *A. thaliana* using the floral dip method (Clough and Bent, 1998). T0 seeds were harvested and sown on MS medium (Murashige and Skoog, 1962) supplemented with 75 mg L^-1^ kanamycin. Three lines (L1, L2, and L3) with the best performance after treatment with 130 mM NaCl and 200 mM mannitol were selected from 59 independent lines, and T3 homozygous lines were generated and used for all subsequent experiments.

### Abiotic Stress Treatments of Transgenic *A. thaliana* Lines

To test the effects of different abiotic stresses on the seed germination rates, approximately 100 seeds from each of the three selected T3 homozygous lines, as well as WT plants, were vernalized for 3 days at 4°C, surface-sterilized and sown on MS medium or MS medium supplemented with 130 mM NaCl or 200 mM mannitol. The germination rate was calculated based on the percentage of seedlings that had reached the cotyledon stage 10 days after sowing (Saleki et al., 1993). All seeds used for the germination analysis were harvested and stored under the same conditions.

To investigate the abiotic stress tolerance, 5-day-old transgenic and WT seedlings grown on MS medium plates were transferred to plates of MS medium, or MS medium supplemented with 130 mM NaCl or 200 mM mannitol. Root lengths and lateral roots number were measured when the seedlings were 20 days old.

Seven-day-old transgenic and WT seedlings grown on MS medium plates were transferred to pots filled with humus and watered well for 3 weeks, during the subsequent week, plants were irrigated at 2 days intervals with 200 mM NaCl or not watered, corresponding to salt and drought treatments, respectively. Plants that were well watered were used as a control. Following the drought treatment, plants were re-watered and survival rates were determined 24 h later. All experiments were repeated three times.

### Measurements of the Chlorophyll Content, Relative Electrolyte Leakage, Malondialdehyde (MDA) and Endogenous ABA Content

Seven-day-old seedlings grown on MS medium were transferred to fresh MS medium, or MS medium supplemented with 130 mM NaCl or 200 mM mannitol, and grown for 1 week before seedlings were harvested. All measurements were repeated three times.

For chlorophyll content measurements, approximately 0.05 g of fresh leaf material was placed in 5 ml of 96% ethanol and incubated at 4°C in the dark overnight. The absorbance of the extracted pigments was measured at 665 and 649 nm using a spectrophotometer (Hitachi Limited, Tokyo, Japan) and the chlorophyll content was calculated as previously described (Zhang H. et al., 2012).

Relative electrolyte leakage was measured as previously described (Cao et al., 2007). Seedlings were vacuum-infiltrated with deionized water for 20 min and after 2 h the conductivity (C1) of the solutions was determined using a conductivity detector. Subsequently, the seedlings were boiled for 20 min in deionized water and cooled to room temperature. The conductivity (C2) of the solution was then determined and the C1 to C2 (C1/C2) ratio was calculated and used as a measure of the relative electrolyte leakage. MDA content was measured as previously described (Heath and Packer, 1968). ABA content was measured using an enzyme-linked immunosorbent assay (ELISA), as previously described (Yang et al., 2001).

### Measurement of the Water Loss Rate

To determine the water loss rate, 10 leaves were detached from 4-week-old transgenic and WT plants and immediately weighed. The samples were then placed on dry filter paper at a relative humidity of 40–45% at room temperature and weighed over a time course. The water loss rate was calculated as previously described (Guo et al., 2015).

### Detection of Reactive Oxygen Species (ROS) and Activity Assays of Antioxidant Enzymes

A histochemical staining procedure was used to detect superoxide and hydrogen peroxide *in situ*, as described in our previously report (Guo et al., 2015). Rosette leaves from 5-week-old transgenic and WT plants that had either been subjected to 200 mM NaCl or drought treatments for 1 week, as well as the corresponding non-treated controls, were infiltrated in 1 mg/ml DAB solution for 8 h or 6 mM NBT for 2 h. Then, the chlorophyll was cleared at 80°C in 80% (v/v) ethanol for 2 h and samples were embedded in 10 % (v/v) glycerol for observations (Kotchoni et al., 2006; Kim et al., 2011). The activities of the antioxidant enzymes in the leaves, including superoxide dismutase (SOD, EC 1.15.1.1), catalase (CAT EC 1.11.1.6) and peroxidase (POD EC 1.11.1.7), were extracted from 0.5 g leaves from abiotic stress treated plants as well as control plants, and measured as described by Tu et al. (2016).

### Measurement of Stomatal Aperture in Response to ABA Treatment

Stomatal aperture assays were performed essentially as described by Pei et al. (1997). Briefly, A. thaliana leaves from 4-week-old plants were incubated in buffer containing 10 mM KCl, 50 μM CaCl_2_, and 10 mM MES/KOH (pH 6.15). To induce stomatal opening, the leaves were first incubated in the light for 2 h and then treated with 1 or 5 μM ABA. Stomatal apertures were observed after 2 h under a microscope (BX53, Olympus, Japan) and recorded as the ratio of stomatal width to length. All assessments were carried out in triplicate.

### Inoculation of *A. thaliana* With Pathogenic Bacteria

Four-week-old T3 transgenic and WT plants were inoculated with *G. cichoracearum*, and visual scoring of disease reaction phenotypes and a spore count of leaves were performed 120 hpi, as previously described (Xiao et al., 2005; Li et al., 2010a). Leaf samples collected 0, 24, 72, and 120 hpi were used to determine the expression profiles of disease resistance related genes.

A *B. cinerea* conidial suspension (1.5 × 10^6^ conidia/ml) was used to inoculate the plants, as previously described (Wan et al., 2015). Detached leaves were used for morphological observation, lesion diameter analysis, and expression analysis of defense related genes, as previously described (Guo et al., 2016). Samples used for morphological observation and lesion diameter analysis were photographed and measured at 72 hpi, while leaves used to analyze the expression of defense related genes were collected at 0, 12, 24, and 48 hpi.

### Gene Expression Analysis by Quantitative Real-Time RT-PCR

Total RNA was extracted from *A. thaliana* leaves using an RNA prep plant kit (Tiangen Biotech., China) following the manufacturer’s protocols. First-strand cDNA was synthesized using PrimeScriptTMRTase (TaKaRa Biotechnology) according to the manufacturer’s instructions. Quantitative real-time RT-PCR (qRT-PCR) analysis was conducted using SYBR green (TaKaRa Biotechnology) and an IQ5 real-time RT-PCR instrument (Bio-Rad, Hercules, CA, United States) with the following thermal profile: 95°C for 30 s, 40 cycles of 95°C for 5 s, and 60°C for 30 s. Each reaction was performed in triplicate for each of the three biologically replicated sets of cDNA samples. To perform the melt-curve analysis, the following program was added after 40 PCR cycles: 95°C for 15 s, followed by a constant increase from 60 to 95°C. A. thaliana *Actin 1* (TAIR^3^: AT2G37620) was used as the reference gene. Primers used for qRT-PCR are listed in **Supplementary Table S1**. Relative expression levels were analyzed with the IQ5 software (Bio-Rad, Hercules, CA, United States) using the Normalized Expression Method.

### Statistical Analysis

All experiments were performed using three biological replicates, with each biological replicate having three technical replicates. Data analysis and plotting were performed using Microsoft Excel (Microsoft Corporation, United States) and Sigma plot (v. 10.0. Systat, Inc., Point Richmond, CA, United States). Significant differences were assessed through paired t-test using the SPSS Statistics 17.0 software (IBM China Company Ltd., Beijing, China).

## Results

### *VlWRKY3* Cloning and Sequence Analysis

Based on the *VvWRKY3* (GSVIVT01010525001) cDNA sequence from the Grape Genome Database (12×)^4^, the *WRKY3* ORF from ‘Kyoho’ was obtained. The *VlWRKY3* coding sequence (CDS) of 570 bp encoded a 190 amino acid protein, and both the CDS sequence and the corresponding deduced amino acid sequence shared 100 % identity to the *V. vinifera* homolog. A phylogenetic tree was produced through analysis the VlWRKY3 protein sequence and its orthologs from a range of plant species, and two clades were contained in the phylogenetic tree, each of which contained sequences from monocotyledonous or dicotyledonous species (**Supplementary Figure S1A**). The *Nicotiana tomentosiformis* protein, XP_009615508.1, shared 54% identity to the VlWRKY3 proteins. The sequence identity between VlWRKY3 and the other proteins in the analysis ranged from 39 to 53%. What’s more, we observed significant sequence conservation within the WRKY domain regions among the sequences, and the sequence logos indicated the level of conservation in the WRKY domain of WRKY proteins at each residue position (**Supplementary Figure S1B**). A total of 48 of the conserved amino acids were identical among all the analyzed WRKY proteins, while there was varying levels of conservation amongst the residues located in other positions (**Supplementary Figure S1B**).

### Generation of 35S::*VlWRKY3* Transgenic *A. thaliana* Lines

To investigate the potential role of *VlWRKY3* in response to abiotic and biotic stresses, *VlWRKY3* was introduced into the *A. thaliana* WT, generating 59 *35S::VlWRKY3* lines. Three lines (L1, L2, L3) were selected to assay for stress tolerance. The transcript levels of *VlWRKY3* in the transgenic lines were evaluated by qRT-PCR (**Supplementary Figure S2A**).

qRT-PCR was also used to examine the expression profiles of *VlWRKY3* in the three transgenic lines post abiotic stress treatments, or *G. cichoracearum* and *B. cinerea* inoculation. Results showed that its expression levels were increased upon exposure to salt and drought treatments, as well as *G. cichoracearum* inoculation (**Supplementary Figures S2B,C**). And its expression levels increased at 12 hpi with *B. cinerea*, and decreased at 48 hpi when compared to expression at 0 hpi (**Supplementary Figure S2D**).

### Effect of Osmotic Stress on Seed Germination and Root Growth in 35S::*VlWRKY3* Transgenic *A. thaliana* Lines

**FIGURE 1:**
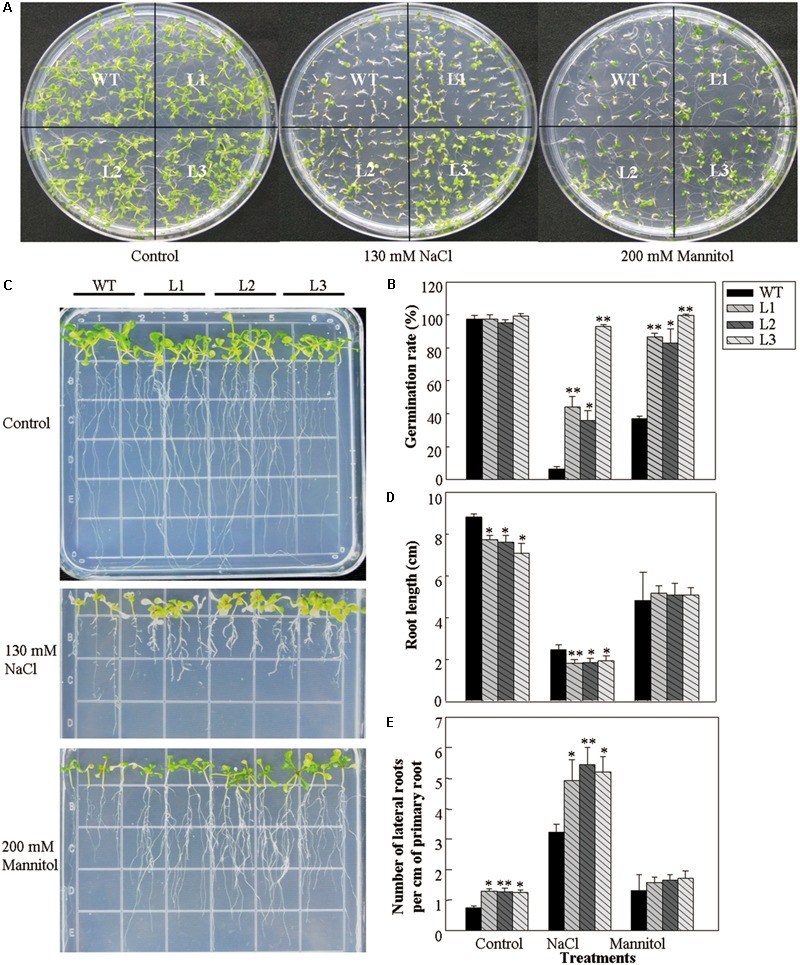
Effect of osmotic stress on seed germination and post-germination growth of WT and *VlWRKY3* transgenic *Arabidopsis thaliana* lines. **(A)** WT and transgenic seedlings 10 days after seeds were sown on Murashige and Skoog (MS) medium or MS basal medium supplemented with 130 mM NaCl and 200 mM mannitol. **(B)** Germination rates of seeds sown on MS, MS + 130 mM NaCl, or MS + 200 mM mannitol plates. Each data point is the mean of three replicates of 100–120 seeds. **(C)** Seedlings at 15 days after transfer to MS, MS + 130mM NaCl, or MS + 200 mM mannitol plates. Seedlings were 5-days-old at the time of transfer. **(D,E)** Root length **(D)** and lateral root number **(E)** of WT and transgenic lines after 15 days treatment with or without 130 mM NaCl and 200 mM mannitol. Each data point is the mean of three replicates of 20–30 seedlings. The error bars indicate the SD. Asterisks indicate statistical significance (^∗^0.01 < *P* < 0.05, ^∗∗^*P* < 0.01, Student’s *t* test) of differences between transgenic lines and WT seedlings.

The effect of *VlWRKY3* on osmotic stress tolerance was tested through comparing the seed germination rates and root growth of *35S::VlWRKY3* transgenic lines and WT. Seeds from *35S::VlWRKY3* transgenic lines and WT were sown on MS basal medium containing NaCl and mannitol to evaluate the response to salt stress and drought stress, respectively. Under normal conditions, almost all of the seeds from both the *35S::VlWRKY3* transgenic lines and WT germinated. However, comparison of germination rates revealed that transgenic seeds exhibited 27–86% higher than that of WT on MS medium containing NaCl or mannitol (**Figures 1A,B**). In addition, the *35S::VlWRKY3* seedlings had shorter roots and higher number of lateral roots per cm of primary root than those of WT when grown on MS basal medium, and MS medium containing NaCl. And there was no difference between the WT and *35S::VlWRKY3* seedlings in the root lengths and number of lateral roots per cm of primary root when both of them were grown on MS medium containing mannitol (**Figures 1D,E**).

### 35S::*VlWRKY3* Transgenic Lines Have Improved Physiological Traits Associated With Osmotic Stress Tolerance

**FIGURE 2:**
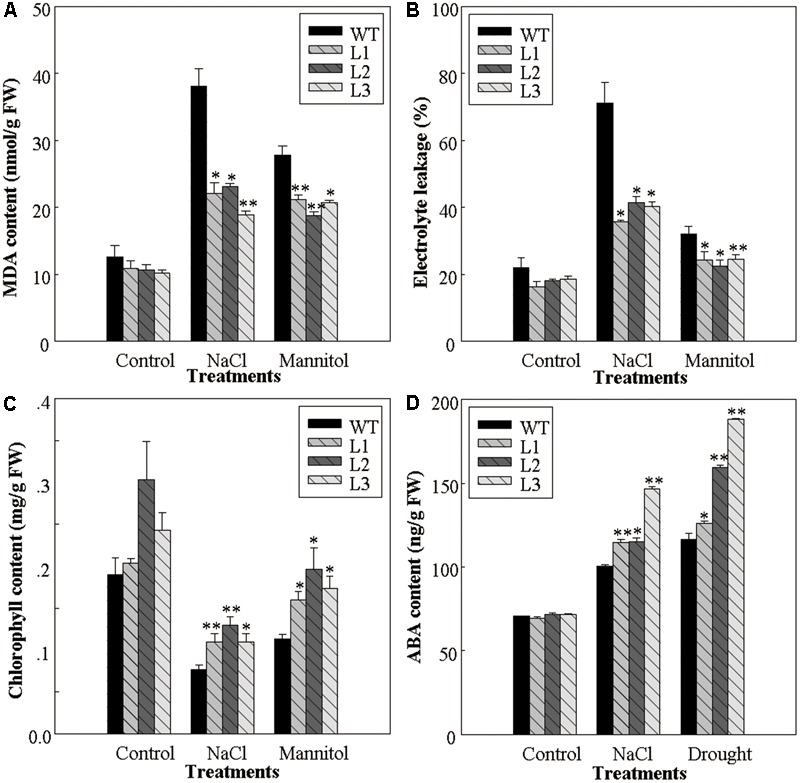
Physiological changes associated with osmotic stress response in WT and *VlWRKY3* transgenic *Arabidopsis thaliana* seedlings. **(A,C,D)** Malondialdehyde (MDA) content **(A)**, Chlorophyll content **(C)** and abscisic acid (ABA) content **(D)** in the leaves of WT and transgenic seedlings grown with or without osmotic stress, respectively. **(B)** Relative electrolyte leakage from detached leaves of WT and transgenic seedlings grown with or without exposure to osmotic stress. In all cases, data values represent means ± SD from three independent experiments. Asterisks indicate statistical significance (^∗^0.01 < *P* < 0.05, ^∗∗^*P* < 0.01, Student’s *t*-test) of differences between transgenic lines and WT seedlings.

The improved osmotic stress tolerance of *35S::VlWRKY3* transgenic seedlings was correlated with changes in several physiological parameters, such as the MDA content, relative electrolyte leakage, and the chlorophyll content, as well as the endogenous ABA content. When grown on MS basal medium, the MDA contents, as well as the relative electrolyte leakage of the *35S::VlWRKY3* lines and the WT were similar. However, the MDA contents of transgenic lines decreased by 44 and 27% when compared to the WT after NaCl and mannitol treatments, respectively (**Figure 2A**), and the relative electrolyte leakage of *35S::VlWRKY3* lines were 45 and 26% lower than those of the WT, respectively (**Figure 2B**). It was also observed that the chlorophyll contents of the leaves of the *35S::VlWRKY3* lines were significantly higher than those of WT corresponding after treatments of NaCl and mannitol treatments (**Figure 2C**), as was also the case for endogenous ABA levels 7 days post the same stress treatments (**Figure 2D**).

### 35S::*VlWRKY3* Transgenic *A. thaliana* Plants Have Increased Tolerance to Salt and Drought Stress

**FIGURE 3:**
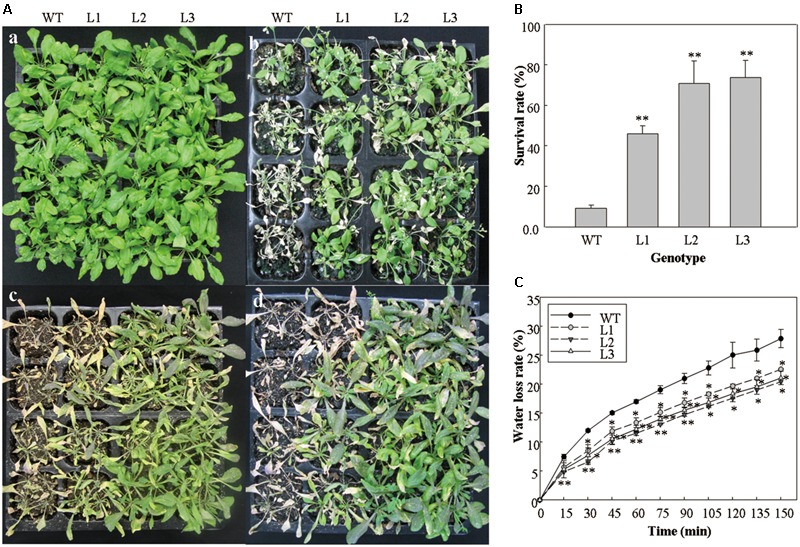
Performance of WT and *VlWRKY3* transgenic *Arabidopsis thaliana* plants under normal growth and abiotic stress conditions. **(A)** Representative images of 5-week-old potted WT and transgenic plants under normal growth and abiotic stress conditions. **(a)** WT and transgenic plants grown for 5 weeks under normal condition. **(b)** Five-week-old WT and transgenic plants that were treated with 200 mM NaCl for 7 days. **(c)** Five-week-old WT and transgenic plants that were deprived of water for 7 days. **(d)** WT and transgenic plants that were deprived of water for 7 days and then re-watered. **(B)** Survival rates of WT and transgenic lines 24 h after re-watering. Each data point is the mean of three replicates of 32 plants. **(C)** Water loss rate of detached leaves of WT and transgenic plants. Each data point is the mean of three replicates of 10 detached leaves. In **(B,C)**, the error bars indicate the SD, Asterisks indicate statistical significance (^∗^0.01 < *P* < 0.05, ^∗∗^*P* < 0.01, Student’s *t*-test) of differences between transgenic lines and WT.

In order to evaluate the tolerance capacity of salt and drought stresses, the performance of 5-week-old *35S::VlWRKY3* transgenic lines and WT that had been subjected to abiotic stresses for 1 week was also investigated. The *35S::VlWRKY3* transgenic lines and the WT plants under the normal growth conditions exhibited no evident differences (**Figure 3A-a**). However, most WT leaves became chlorotic after treatment with 200 mM NaCl for 7 days, while the transgenic lines still remained green and phenotypically normal under the same conditions (**Figure 3A-b**). All WT plants displayed significant wilting and water loss after 7 days of withholding water, but the *35S::VlWRKY3* transgenic lines only showed mild wilting (**Figure 3A-c**). All of the plants were re-watered after 7 days of water deprivation, and almost all of the WT plants died, whereas 46–74% of the *35S::VlWRKY3* transgenic lines survived after 1 d of re-watering (**Figures 3A-d,B**).

Four-week-old detached rosette leaves of WT and *VlWRKY3* transgenic lines were used to assess the water loss rate. Results showed that the water loss rates of leaves from the three *35S::VlWRKY3* transgenic lines were lower than the WT plants at most time points (**Figure 3C**).

**FIGURE 4:**
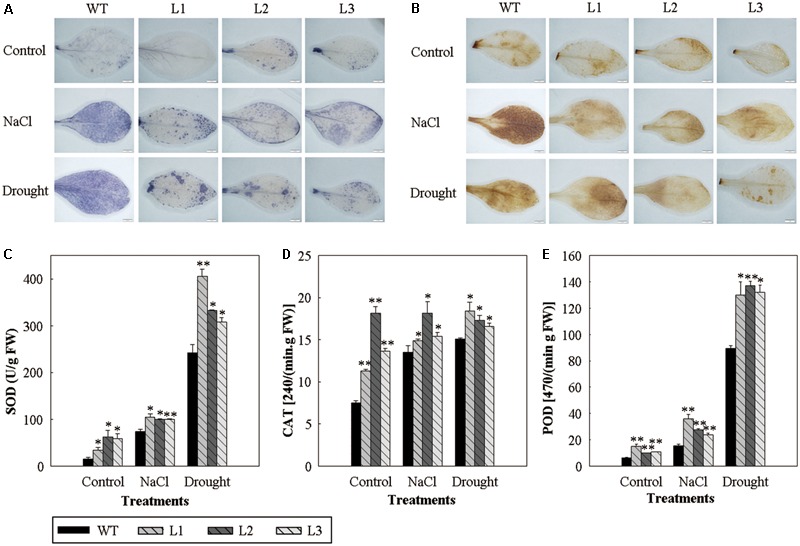
Reactive oxygen species levels and activity assays of activated oxygen scavenging enzymes in WT and *VlWRKY3* transgenic *Arabidopsis thaliana* plants under normal growth and abiotic stress conditions. **(A,B)** Histochemical staining assay of O_2_^-^ and H_2_O_2_ accumulation with NBT **(A)** and DAB **(B)**, in WT and transgenic leaves following normal growth and abiotic stress conditions. The experiment was repeated 3 times, and 5–10 leaves were stained in each experiment. **(C–E)** Activity of SOD **(C)**, CAT **(D)** and POD **(E)** in the leaves of WT and transgenic plants under normal growth and abiotic stress conditions. Data values represent means ± SD from three independent experiments. Asterisks indicate statistical significance (^∗^0.01 < *P* < 0.05, ^∗∗^*P* < 0.01, Student’s *t*-test) of differences between transgenic lines and WT plants.

In order to determine whether the production of ROS in the *35S::VlWRKY3* transgenic plants was affected by abiotic stress, the leaves of transgenic plants and WT that had been subjected to abiotic stresses were histochemically stained with NBT and DAB to determine the levels of O_2_^-^ and H_2_O_2_, respectively. Results showed that the leaves of the WT plants accumulated much more ROS than those of the *35S::VlWRKY3* transgenic plants under abiotic stress conditions (**Figures 4A,B**).

To test the antioxidant enzymes-mediated ROS detoxification in the *35S::VlWRKY3* transgenic plants, we determined the enzymatic activities of SOD, CAT, and POD after salt and drought stress treatments. The three *35S::VlWRKY3* transgenic plants possessed higher activities of the three antioxidant enzymes than those corresponding WT with or without abiotic stress treatments (**Figures 4C–E**), which was consistent with the lower accumulation of ROS in the transgenic plants.

### 35S::*VlWRKY3* Transgenic *A. thaliana* Plants Have Increased Sensitivity of Stomata After ABA Treatment

**FIGURE 5:**
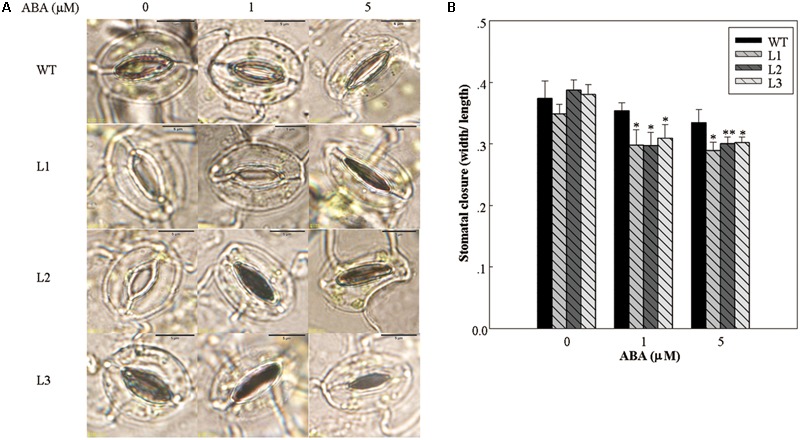
Stomatal closure in response to abscisic acid (ABA) treatment in WT and *VlWRKY3* transgenic *Arabidopsis thaliana* plants. **(A)** Comparison of stomatal apertures in response to different concentrations of exogenous ABA in WT and transgenic plants. **(B)** Stomatal aperture width to length ratios following treatment with different concentrations of exogenous ABA. Data values represent means ± SD from three independent experiments. Asterisks indicate statistical significance (^∗^0.01 < *P* < 0.05, ^∗∗^*P* < 0.01, Student’s *t*-test) of differences between transgenic lines and WT plants.

Water loss from leaves is strongly influenced by stomatal regulation, which is in turn affected by the concentration of ABA in the leaves (Yan et al., 2014). The largest water loss was observed in WT, whereas 35S::*VlWRKY3* transgenic plants exhibited lower water loss (**Figure 3C**). In addition, we compared the stomatal apertures from the leaves of both transgenic and WT plants that has been treated with different concentrations of exogenous ABA. As shown in **Figure 5**, stomatal apertures of the *35S::VlWRKY3* transgenic lines were significantly lower than that of WT plants when ABA was applied, whereas untreated transgenic and WT plants had similar, and relatively high stomatal apertures (**Figure 5**).

### 35S::*VlWRKY3* Transgenic *A. thaliana* Plants Have Altered Expression Levels of Abiotic Stress Responsive Genes

**FIGURE 6:**
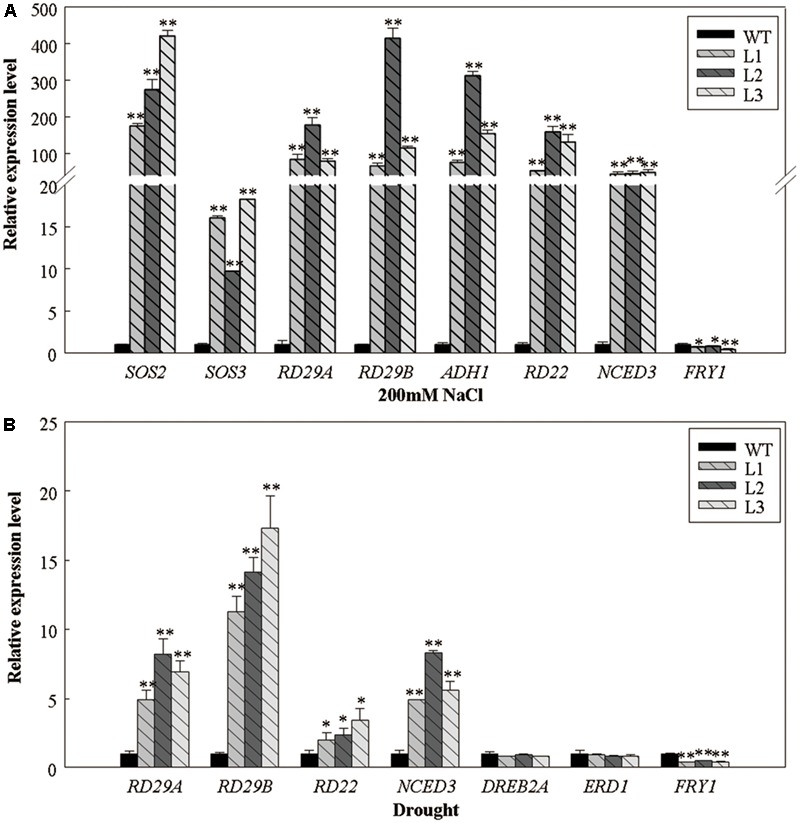
Expression levels of abiotic stress-responsive genes in WT and *VlWRKY3* transgenic *Arabidopsis thaliana* plants following exposure to abiotic stresses. **(A)** The relative expression levels of salt stress-responsive genes in WT and transgenic plants treated with 200 mM NaCl for 7 days. **(B)** The relative expression levels of drought stress-responsive genes in WT and transgenic plants deprived of water for 7 days. Relative gene expression levels were analyzed using quantitative real-time RT-PCR. Bars represent the mean ± SD of three independent experiments. Asterisks indicate statistical significance (^∗^0.01 < *P* < 0.05, ^∗∗^*P* < 0.01, Student’s *t*-test) of differences between transgenic lines and WT plants.

We examined the expression of several abiotic stress-responsive genes in 5-week-old *35S::VlWRKY3* transgenic *A. thaliana* plants that had been subjected to salt or drought stress treatments for 1 week (**Figure 6**). The expression levels of *RD29A, RD29B, RD22*, and *ADH1* appeared more abundant in the *35S::VlWRKY3* transgenic plants than those in the WT after salt and drought treatments (**Figure 6**). In contrast, *FRY1*, which supresses the ABA signaling pathway (Xiong et al., 2001), was expressed at lower levels upon exposure to abiotic stress (**Figure 6**). *SOS2* and *SOS3*, which involved in the SOS pathway (Zhu, 2002), was up-regulated in the *35S::VlWRKY3* transgenic plants after salt treatment (**Figure 6A**). However, no evident differences were observed in the expression of *DREB2A* and *ERD1* between the *35S::VlWRKY3* transgenic and WT plants post drought treatment (**Figure 6B**). We also evaluated the expression of the ABA biosynthesis associated gene, *NCED3* (Iuchi et al., 2001), after abiotic stress treatments, and its expression levels in the *35S::VlWRKY3* transgenic plants were 5-48 fold greater than in the WT (**Figure 6**).

### 35S::*VlWRKY3* Transgenic *A. thaliana* Plants Have Enhanced Susceptibility to *B. cinerea*

**FIGURE 7:**
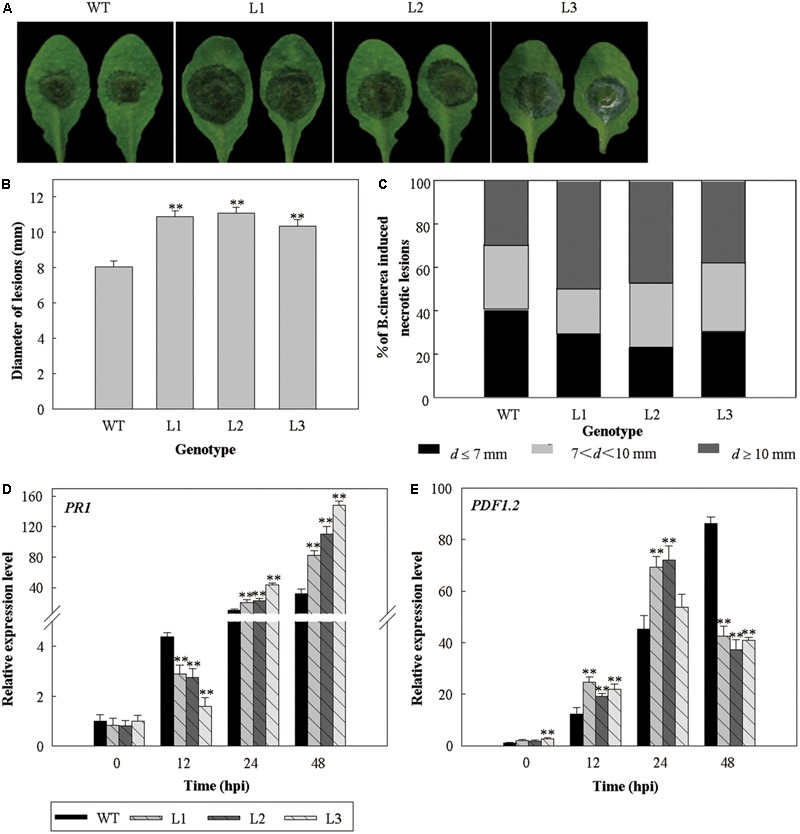
The response of *VlWRKY3* over-expressing *Arabidopsis thaliana* to inoculation with *Botrytis cinerea*. **(A)** The phenotypes of transgenic and WT leaves 3 days after inoculation. **(B)** Average lesion diameters 3 days after inoculation. Data values represent mean ± SD from three independent experiments with at least 50 leaves per sample. Asterisks indicate statistical significance (^∗∗^*P* < 0.01, Student’s *t*-test) between transgenic lines and WT. **(C)** Symptoms 3 days after inoculation were scored by defining three lesion diameter (d) classes: d ≤ 7 mm; 7 < d < 10 mm; d ≥ 10 mm. Data values represent means from three independent experiments with at least 50 leaves per sample. **(D,E)** Relative expression levels of PR1 **(D)** and PDF1.2 **(E)** Relative gene expression levels were determined using quantitative RT-PCR. Bars represent the mean ± SD of three independent experiments. Asterisks indicate statistical significance (^∗^0.01 < *P* < 0.05, ^∗∗^*P* < 0.01, Student’s *t*-test) between transgenic lines and WT.

Since we previously found that the expression levels of *VlWRKY3* was influenced by MeJA and Eth treatments (Guo et al., 2014), we hypothesized that it operates via JA and Eth mediated signaling pathways. It is also generally accepted that JA and Eth act in the necrotrophic pathogen-induced disease resistance response (Oliver and Solomon, 2004). Three dpi with *B. cinerea*, a necrotrophic pathogen, the phenotypes of leaves from the *35S::VlWRKY3* transgenic lines and WT were examined and lesion diameters on the leaves were scored and classified into size categories. The lesions on the *35S::VlWRKY3* transgenic leaves were larger than those on the WT leaves (**Figures 7A,B**), and the proportions of medium and large sized lesions were higher (**Figure 7C**). The expression level of *PR1* was lower at 12 hpi, and higher at 24 and 48 hpi in the *35S::VlWRKY3* transgenic plants compared to the WT (**Figure 7D**), while the expression of *PDF1.2* was up-regulated at 12 and 24 hpi, and down-regulated at 48 hpi in the transgenic plants (**Figure 7E**).

### 35S::*VlWRKY3* Transgenic *A. thaliana* Plants Have Enhanced Resistance to *G. cichoracearum*

**FIGURE 8:**
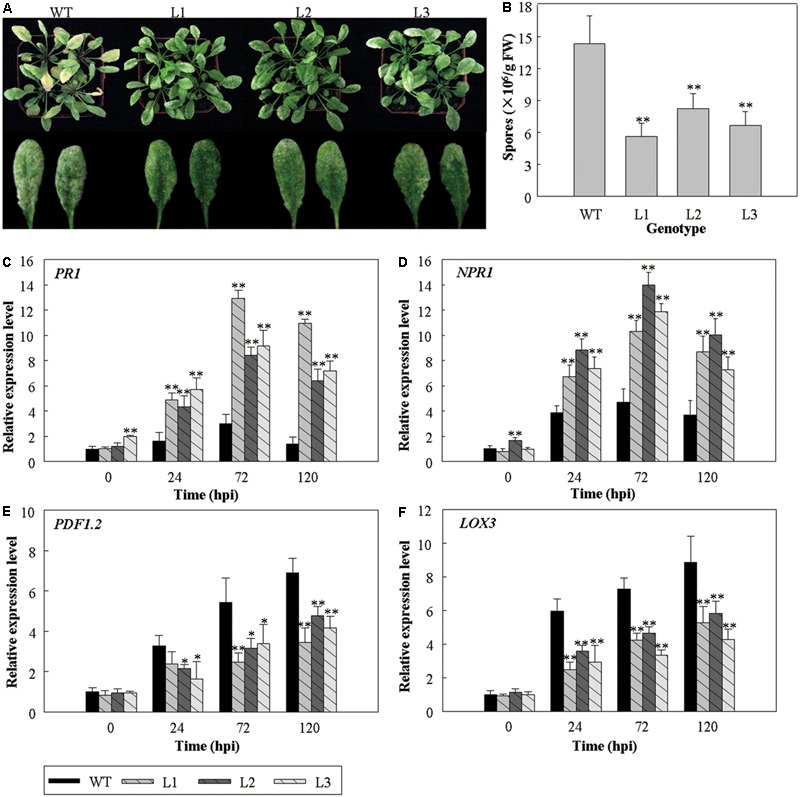
The response of *VlWRKY3* transgenic *Arabidopsis thaliana* to *Golovinomyces cichoracearum* inoculation. **(A)** The phenotype of transgenic and WT plants post infection for 5 days. **(B)** Bacteria numbers per gram of fresh leaf tissue 5 days post infection. **(C–F)** The relative expression levels of *PR1* **(C)**, *NPR1* **(D)**, *PDF1.2* **(E)** and *LOX3* **(F)** in WT and *VlWRKY3* transgenic *A. thaliana* plants following *G. cichoracearum* infection. Relative gene expression levels were analyzed using quantitative RT-PCR. Bars represent the mean values ± SD of three independent experiments. Asterisks indicate statistical significance (^∗^0.01 < *P* < 0.05, ^∗∗^*P* < 0.01, Student’s *t-*test) between transgenic lines and WT.

To further investigate the putative function of *VlWRKY3* in biotrophic pathogens-induced defense responses, the *35S::VlWRKY3* transgenic and WT plants were inoculated with *G. cichoracearum*. Results showed that the *35S::VlWRKY3* transgenic plants were more resistant against *G. cichoracearum* at 5 dpi compared to the WT (**Figure 8A**), and conidia count on *35S::VlWRKY3* transgenic leaves was significantly lower than in the WT plants (**Figure 8B**). The expression patterns of *PR1* and *NPR1* were significantly increased at 24, 72, and 120 hpi in transgenic lines compared to the WT plants, and reached maximum expression at 72 hpi (**Figures 8C,D**). *PDF1.2* and *LOX3* was also up-regulated after *G. cichoracearum* inoculation, and reached a peak at 120 hpi, but their expression levels in the *35S::VlWRKY3* transgenic plants were suppressed when compared to the WT (**Figures 8E,F**).

## Discussion

Members of the WRKY transcription factor family play important roles in diverse plant developmental and physiological processes, including embryogenesis (Lagace and Matton, 2004), seed coat and trichome development (Johnson et al., 2002), leaf senescence (Miao and Zentgraf, 2007; Zhou et al., 2011), as well as various plant abiotic and biotic stress responses (Guo et al., 2014). In grape, 59 WRKY genes have been identified and classified into three main groups (I-III), with the *VlWRKY3* gene belonging to Group IIc. In this study, the gene sequence of *VlWRKY3* from ‘Kyoho’ was isolated. Phylogenetic analysis of the VlWRKY3 sequence, together with orthologs from a range of plant species, revealed a phylogenetic tree with two distinct clades (**Supplementary Figure S1A**), consistent with the WRKY3 sequence evolving substantially after the divergence of dicots and monocots from their last common ancestor (Zhang L.C. et al., 2012).

In a previously study, the expression of *VlWRKY3* was induced post salt and drought stress treatments, as well as after addition of exogenous MeJA and Eth (Guo et al., 2014), suggesting a role in both abiotic and biotic stress responses. This hypothesis was tested through a series of experiments involving transgenic *A. thaliana* plants over-expressing *VlWRKY3*. First of all, it was found that the expression levels of *VlWRKY3* in transgenic *A. thaliana* plants were affected by abiotic and biotic stress treatments (**Supplementary Figures S2B–D**), which would be caused by the influence of other genes that involved in its interaction network. In terms of general osmotic stress, transgenic seeds exhibited significantly increased levels of germination compared to WT seeds (**Figures 1A,B**). Lateral roots contribute to water uptake and facilitate the extraction of nutrients required for growth and development (Zhu et al., 2012). Interestingly, the number of lateral roots per cm of primary root in *VlWRKY3* over-expressing seedlings was higher than in WT, and the roots were shorter than in WT under control and salt stress conditions (**Figures 1C–E**). In addition, the transgenic plants were substantially more tolerant of salt and dehydration than WT plants (**Figures 3A,B**). These findings suggest that *VlWRKY3* is involved in regulating responses to osmotic stress, and may have values as a target for crop improvement.

To investigate the mechanisms by which *VlWRKY3* confers abiotic stress tolerance, we performed several experiments to monitor the physiological changes associated with stress responses. It has been reported that drought and salt stresses can be accompanied by the production of MDA (Levine et al., 1994), which was the marker of lipid peroxidation and therefore of membrane damage. We observed a lower MDA content in *35S::VlWRKY3* transgenic seedlings than in WT after exposure to osmotic stress (**Figure 2A**). It is generally accepted that maintenance of integrity and stability of cell membranes under water stress conditions is a major factor in drought tolerance, and that the degree of cell membrane injury induced by water stress can be assessed through measurements of electrolyte leakage from the cells (Bajji et al., 2002). The levels of electrolyte leakage in the WT seedlings were significantly higher than in any of the transgenic lines under salt and mannitol stress (**Figure 2B**), indicating that the cell membranes of the WT suffered more damage. As a reliable, non-invasive method for monitoring photosynthetic events, chlorophyll fluorescence was used to reflect the physiological status of plants (Strasser et al., 2002), and we observed that the chlorophyll content of *35S::VlWRKY3* transgenic seedlings was significantly higher than in the WT plant after the osmotic stress treatments (**Figure 2C**).

Abscisic acid plays a central role in responses to various abiotic stresses (Fujita et al., 2006), and significant differences were detected in endogenous ABA content between mature *35S::VlWRKY3* transgenic and WT plants that had been exposed to abiotic stress (**Figure 2D**). This was confirmed by the observation that the transcript abundance of *NCED3*, whose expression is often used as an indicator of ABA biosynthesis, was also significantly higher in the transgenic plants than in WT following the abiotic stress treatments (**Figure 6**). What’s more, when plants were subjected to water deficit condition, the increased endogenous ABA content could trigger ROS production to mediate down stream responses in guard cells (Desikan et al., 2004). In this study, we observed that the endogenous ABA content in *35S::VlWRKY3* transgenic lines were much higher than in WT plants (**Figure 2D**), but the ROS levels were lower than in WT plants post drought stress treatment for 7 days (**Figures 4A,B**). A lower width to length ratio in the transgenic plants than in WT following various concentrations of exogenous ABA treatment was also observed (**Figure 5**). It has been reported that the accumulation of excessive levels of ROS can cause oxidative damage to cells and stimulate the activity of antioxidant systems, such as SOD, CAT and POD (Mittler, 2002). And significantly higher activities of SOD, CAT and POD were measured in transgenic plants after normal conditions and salt as well as drought stress for 7 days in the present study (**Figures 4C–E**), suggesting that *35S::VlWRKY3* transgenic plants may be able to scavenge excessive ROS under severe abiotic stress conditions. Thus, it would be speculated that the increased endogenous ABA content triggered ROS production, and then promoted the closure of stomata in transgenic plants, and excessive ROS was scavenged by SOD, CAT and POD whose activities were increased in transgenic plants post drought stress treatment.

Unexpectedly, H_2_O_2_ and O_2_^-^ accumulation in transgenic lines were higher than in WT plants at 48 hpi with *B. cinerea* (**Supplementary Figure S3**), and this would promote the susceptibility of transgenic lines to *B. cinerea* infection and colonization (**Figures 7A–C**). The difference in ROS accumulation between abiotic stress treatments and *B. cinerea* inoculation may be cause by the different mechanisms of *VlWRKY3* in response to abiotic and biotic stress, and the influence of other genes involved in its regulatory network.

We also observed that the expression levels of downstream components of ABA signaling, such as *RD29A, RD29B, RD22*, and *ADH1*, were higher in the *35S::VlWRKY3* transgenic plants, while *FRY1* was down-regulated upon salt and/or drought stress, and this could be caused by enhanced expression of *NCED3* and increased ABA content (**Figure 6**). The expression levels of *SOS2* and *SOS3*, involved in the SOS pathway, was higher in the transgenic plants, indicating a role for *VlWRKY3* in enhancing salt stress tolerance in the transgenic plants (**Figure 8A**). In addition, the expression levels of two ABA-independent genes, *DREB2A* and *ERD1*, were similar in WT and the transgenic plants under drought stress condition (**Figure 6B**), suggesting that the over-expression of *VlWRKY3* does not influence the ABA-independent signaling pathway.

Some WRKY transcription factors have been implicated in the regulation of various biological processes, including pathogen response and hormone signaling (Eulgem and Somssich, 2007). In one of our previous studies, the expression of *VlWRKY3* was induced by MeJA and Eth treatments, but not by SA treatment and powdery mildew infection (Guo et al., 2014). Since the JA and Eth signaling pathways mainly mediate resistance to necrotrophic pathogens, while the SA signaling pathway is involved in resistance to biotrophic pathogens (Chen L.G. et al., 2010), it was speculated that *VlWRKY3* promoted the response to necrotrophic pathogens. To assess the function of *VlWRKY3* in response to different pathogens, we examined the infection phenotypes, as well as the expression profiles of disease resistance related genes post pathogen infection. The *35S::VlWRKY3* transgenic *A. thaliana* plants showed increased susceptibility to *B. cinerea*, a necrotrophic pathogen (**Figures 7A–C**), and the expression of *AtPR1* was inhibited at 12 hpi, but induced at 24 and 48 hpi. In addition, the expression of *AtPDF1.2* increased at 12 and 24 hpi, and decreased at 48 hpi in transgenic plants compared to the WT (**Figures 7D,E**). This indicated that over-expression of *VlWRKY3* inhibits the expression of *AtPR1* and *AtPDF1.2*, at the early stage and later stage of infection, respectively. Since *PDF1.2* was induced by MeJA (Higashi et al., 2008), we inferred that the MeJA signaling was enhanced at an early stage. However, the enhanced MeJA signaling did not increase the resistance of transgenic plants to *B. cinerea* (**Figure 7**). To further investigate the putative function of *VlWRKY3* in defense processes, the three transgenic *A. thaliana* lines and WT were inoculated with *G. cichoracearum*, a biotrophic pathogen. We observed that the transgenic plants were more resistant to *G. cichoracearum* than the WT (**Figures 8A,B**), and expression of *PR1* and *NPR1* increased, while *PDF1.2* and *LOX3* expression was lower in the transgenic plants than in WT (**Figures 8C–F**).

The results that *35S::VlWRKY3* transgenic plants respond to necrotrophic and biotrophic pathogens infection was surprising, since this would indicate an interaction of SA and MeJA signaling pathways. The function of *VlWRKY3* may therefore be affected by other genes, a hypothesis that will be the focus of further studies.

## Conclusion

Based on the performance of *VlWRKY3* over-expressing plants, it was demonstrated that this gene had positive functions in tolerance to salt and drought stresses. Moreover, it was speculated that *VlWRKY3* was involved in response to different pathogen infections. Further studies are needed to investigate the mechanisms of action of *VlWRKY3* in response to abiotic and biotic stresses.

## Author Contributions

XpW and RG designed the study. RG, HQ, and JZ contributed to the experiment. XaW and MT performed the data analysis. CG did the quantitative real-time PCR. RW and ZL assisted with the interpretation of the results. ZL and XpW overall provided guidance on the whole study. RG and XpW wrote the manuscript. All authors approved the final manuscript.

## Conflict of Interest Statement

The authors declare that the research was conducted in the absence of any commercial or financial relationships that could be construed as a potential conflict of interest.

## Footnotes

1 http://blast.ncbi.nlm.nih.gov/Blast.cgi

2 http://meme.sdsc.edu/meme/intro.html

3 http://www.arabidopsis.org/

4 http://www.genoscope.cns.fr

## Supplementary Material

FIGURE S1 Phylogenetic relationships and sequence logos for the WRKY domains of the VlWRKY3 protein and its homologs in other plant species. The accession numbers for each sequence are the following: *Pyrus* × *bretschneideri* (XP_009343134.1), *Malus domestica* (XP_008389898.1), *Prunus persica* (XP_007226197.1), *Citrus clementina* (XP_006437986.1), *Populus trichocarpa* (XP_002304741.2), *Solanum lycopersicum* (XP_004239240.1), *Vitis vinifera* (XP_002275576.1), *Nicotiana tomentosiformis* (XP_009615508.1), *Ricinus communis* (XP_002509495.1), *Arabidopsis thaliana* (NP_196812.1), *Cucumis sativus* (XP_004148012.1), *Sorghum bicolor* (XP_002449513.1), *Zea mays* (XP_008678126.1), *Seteria italica* (XP_004979296.1), *Oryza sativa* (DAA05137.1), *Brachypodium distachyon* (XP_003577566.1), *Triticum aestivum* (ABN43184.1). **(A)** Phylogenetic relationships of the VlWRKY3 protein and its homologs. The divergence of the clades between the monocots and dicots is indicated by the dotted lines. **(B)** The sequence logos for the WRKY domain of VlWRKY3 and its homologues. The overall height of each stack indicates the conservation of the protein sequence at that amino acid position, and the height of letters within each stack represents the relative frequency of the corresponding amino acid.

FIGURE S2 *VlWRKY3* expression and abiotic and biotic-responsiveness in transgenic *Arabidopsis thaliana*. **(A)** The expression level of *VlWRKY3* in 4-week-old seedling leaves from untreated WT and three independent transgenic lines (L1, L2, and L3) over-expressing the *VlWRKY3*. Asterisks indicate statistical significance (^∗∗^*P* < 0.01, Student’s *t*-test) in comparison with WT. **(B)** The expression level of *VlWRKY3* in WT and transgenic lines that were subjected to 200 mM NaCl or drought treatments for 7 days, respectively. Asterisks indicate statistical significance (^∗∗^*P* < 0.01, Student’s *t*-test) of differences between the abiotic treatments and control conditions in different lines. **(C)** The expression level of *VlWRKY3* following *Golovinomyces cichoracearum* infection. Asterisks indicate statistical significance (^∗^0.01 < *P* < 0.05, ^∗∗^*P* < 0.01, Student’s *t*-test) of differences between the expression level at 24, 72, 120, and 0 h after infection in the different lines. **(D)** The expression level of VlWRKY3 following *Botrytis cinerea* infection. Asterisks indicate statistical significance (^∗^0.01 < *P* < 0.05, ^∗∗^*P* < 0.01, Student’s *t*-test) of differences between the expression level at 12, 24, 48, and 0 h after infection in the different lines. Data values represent means ± SD from three independent experiments.

FIGURE S3 ROS levels in WT and *VlWRKY3* transgenic *Arabidopsis thaliana* plants post *Botrytis cinerea* infection 48 h. **(A,B)** Histochemical staining assay of H_2_O_2_ and O_2_^-^ accumulation with nitro blue tetrazolium (NBT) **(A)** and diaminobenzidine (DAB) **(B)** in WT and transgenic leaves. The experiment was repeated 3 times with 5–10 leaves.

TABLE S1 Stress-response genes selected from the TAIR database, and gene-specific primers used for qRT-PCR.

## Abbreviations

ABA: abscisic acid
CAT: catalase
CDS: coding sequence
d: diameter
DAB: diaminobenzidine
dpi: days post inoculation
ET: ethylene
hpi: hours post inoculation
MDA: malonyl dialdehyde
MeJA: methyl jasmonate
NBT: nitro blue tetrazolium
ORF: open reading frame
*pad4*: *Phytoalexin deficient 4*
POD: peroxidase
qRT-PCR: quantitative real-time PCR
ROS: reactive oxygen species
SOD: superoxide dismutase
SOS: salt overly sensitive
WT: wild-type
