# Supplementary figures and images for: The Grape VlWRKY3 Gene Promotes Abiotic and Biotic Stress Tolerance in Transgenic Arabidopsis thaliana
Source: Front Plant Sci. 2018 Apr 25;9:545. doi: 10.3389/fpls.2018.00545 (PMC5996931; doi:10.3389/fpls.2018.00545)

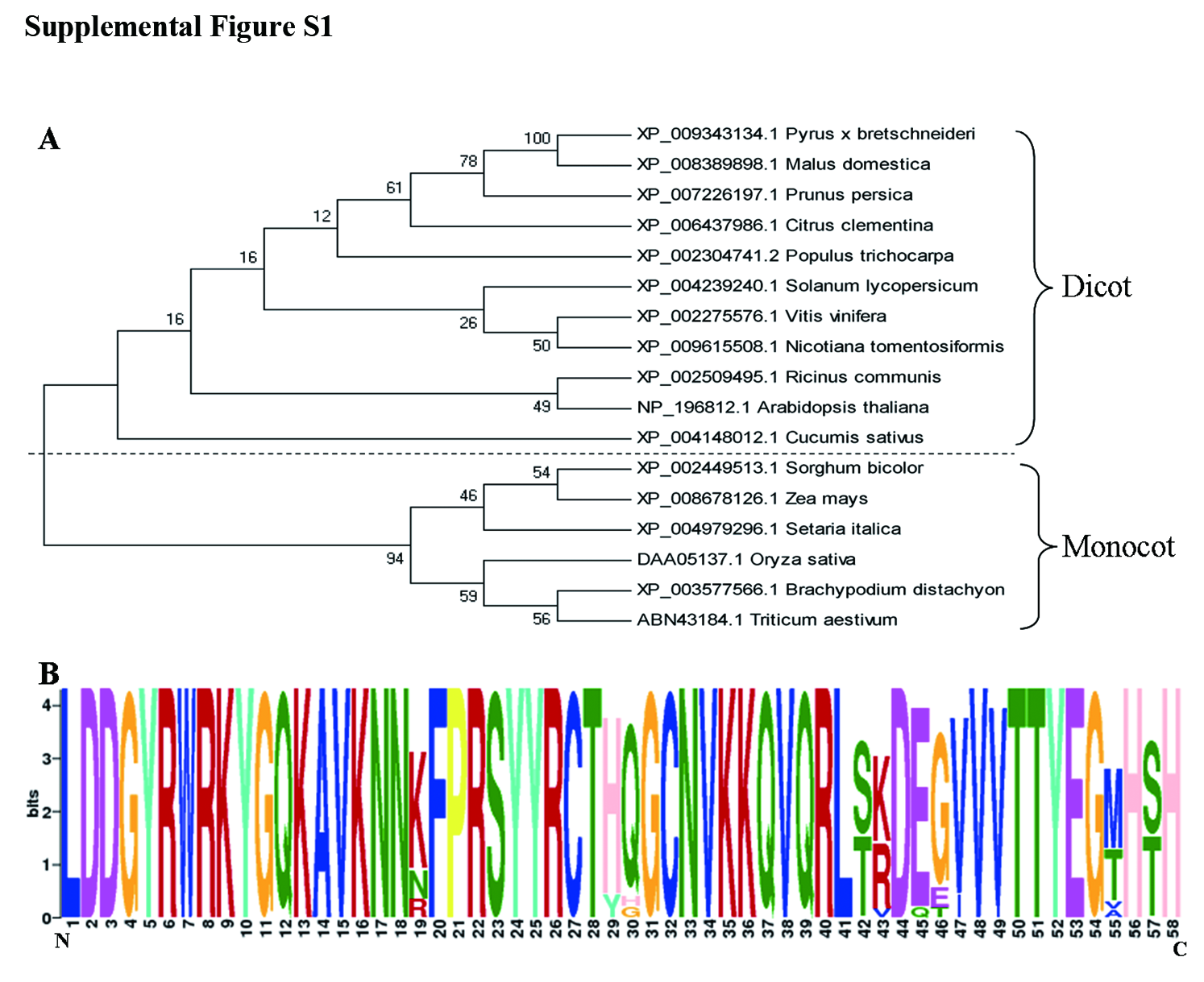

Supplement: FIGURE S1 — Phylogenetic relationships and sequence logos for the WRKY domains of the VlWRKY3 protein and its homologs in other plant species. The accession numbers for each sequence are the following: Pyrus × bretschneideri (XP_009343134.1), Malus domestica (XP_008389898.1), Prunus persica (XP_007226197.1), Citrus clementina (XP_006437986.1), Populus trichocarpa (XP_002304741.2), Solanum lycopersicum (XP_004239240.1), Vitis vinifera (XP_002275576.1), Nicotiana tomentosiformis (XP_009615508.1), Ricinus communis (XP_002509495.1), Arabidopsis thaliana (NP_196812.1), Cucumis sativus (XP_004148012.1), Sorghum bicolor (XP_002449513.1), Zea mays (XP_008678126.1), Seteria italica (XP_004979296.1), Oryza sativa (DAA05137.1), Brachypodium distachyon (XP_003577566.1), Triticum aestivum (ABN43184.1). (A) Phylogenetic relationships of the VlWRKY3 protein and its homologs. The divergence of the clades between the monocots and dicots is indicated by the dotted lines. (B) The sequence logos for the WRKY domain of VlWRKY3 and its homologues. The overall height of each stack indicates the conservation of the protein sequence at that amino acid position, and the height of letters within each stack represents the relative frequency of the corresponding amino acid. [file Image_1.tif]

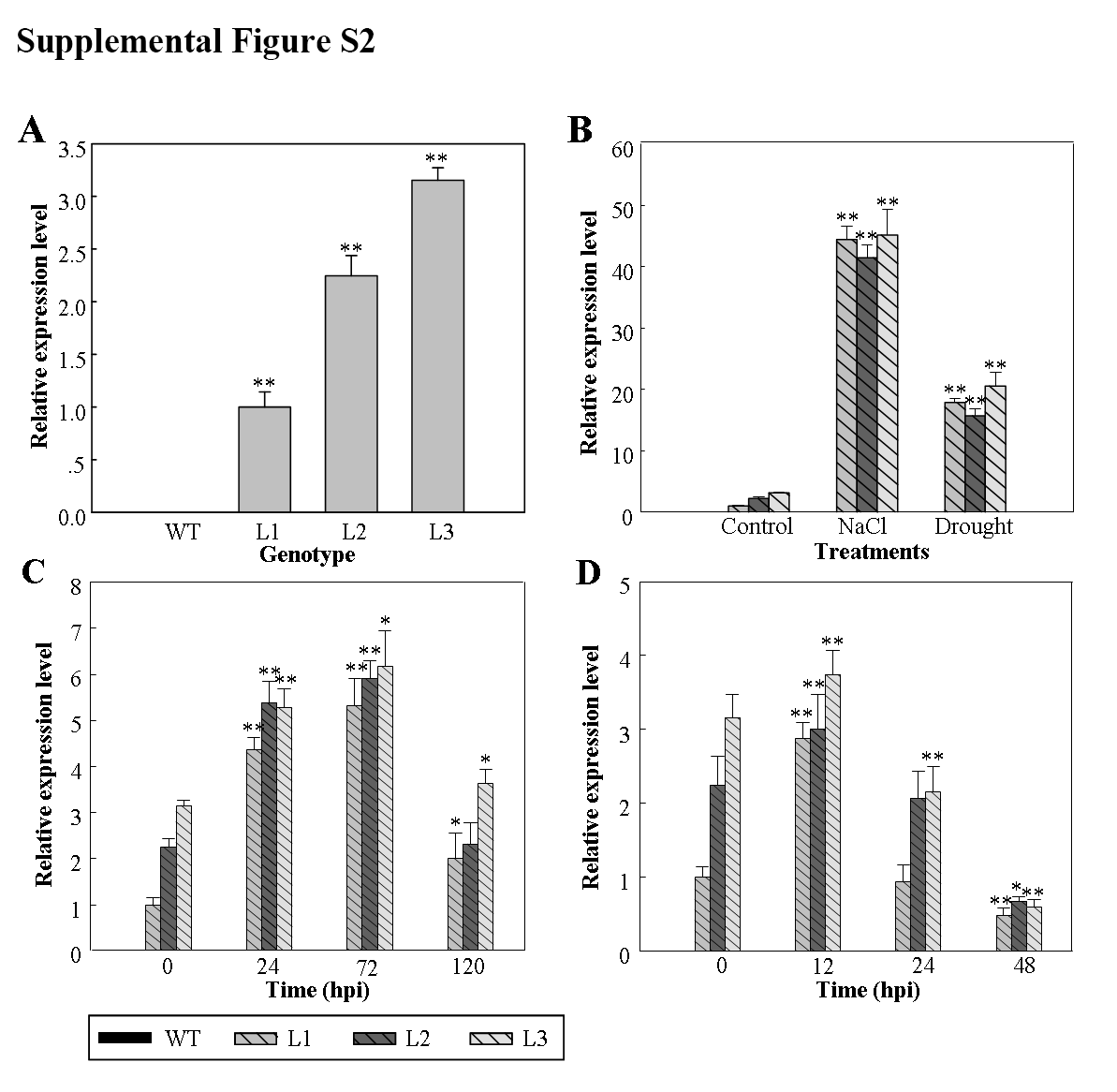

Supplement: FIGURE S2 — VlWRKY3 expression and abiotic and biotic-responsiveness in transgenic Arabidopsis thaliana. (A) The expression level of VlWRKY3 in 4-week-old seedling leaves from untreated WT and three independent transgenic lines (L1, L2, and L3) over-expressing the VlWRKY3. Asterisks indicate statistical significance (∗∗P < 0.01, Student’s t-test) in comparison with WT. (B) The expression level of VlWRKY3 in WT and transgenic lines that were subjected to 200 mM NaCl or drought treatments for 7 days, respectively. Asterisks indicate statistical significance (∗∗P < 0.01, Student’s t-test) of differences between the abiotic treatments and control conditions in different lines. (C) The expression level of VlWRKY3 following Golovinomyces cichoracearum infection. Asterisks indicate statistical significance (∗0.01 < P < 0.05, ∗∗P < 0.01, Student’s t-test) of differences between the expression level at 24, 72, 120, and 0 h after infection in the different lines. (D) The expression level of VlWRKY3 following Botrytis cinerea infection. Asterisks indicate statistical significance (∗0.01 < P < 0.05, ∗∗P < 0.01, Student’s t-test) of differences between the expression level at 12, 24, 48, and 0 h after infection in the different lines. Data values represent means ± SD from three independent experiments. [file Image_2.tif]

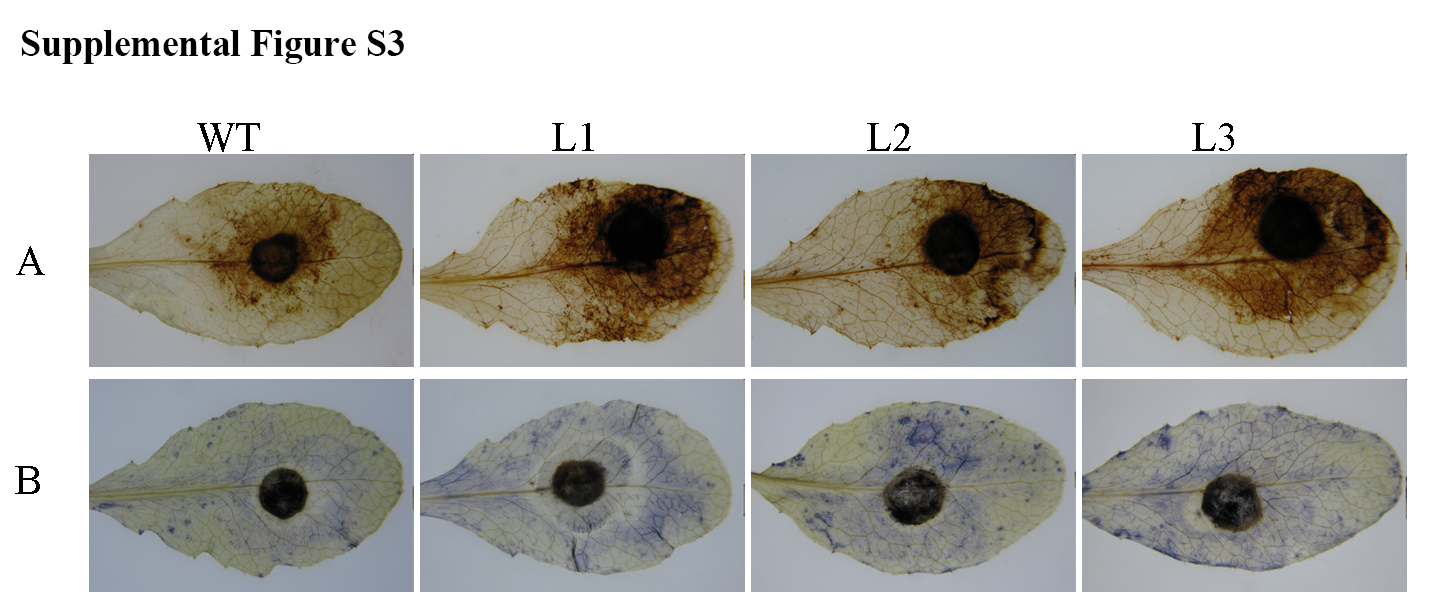

Supplement: FIGURE S3 — ROS levels in WT and VlWRKY3 transgenic Arabidopsis thaliana plants post Botrytis cinerea infection 48 h. (A,B) Histochemical staining assay of H2O2 and O2- accumulation with nitro blue tetrazolium (NBT) (A) and diaminobenzidine (DAB) (B) in WT and transgenic leaves. The experiment was repeated 3 times with 5–10 leaves. [file Image_3.tif]
