# Supplementary material for: The Grape VlWRKY3 Gene Promotes Abiotic and Biotic Stress Tolerance in Transgenic Arabidopsis thaliana
Source: Front Plant Sci. 2018 Apr 25;9:545. doi: 10.3389/fpls.2018.00545 (PMC5996931; doi:10.3389/fpls.2018.00545)
Supplement: TABLE S1 — Stress-response genes selected from the TAIR database, and gene-specific primers used for qRT-PCR. [file Table_1.doc]

Supplemental Table S1 Stress-response genes selected from the TAIR database, and gene-specific primers used for qRT-PCR.

| Genes | Forwards primers (5'-3') | Reverse primers (5'-3') | Locus_tag |
| --- | --- | --- | --- |
| *VvWRKY3* | AGTTCCCGCCTCAGACATC | TTTTGCCCATACTTCCTCC | XM_002275540.3 |
| *VvActin1* | GATTCTGGTGATGGTGTGAGT | GACAATTTCCCGTTCAGCAGT | AY680701 |
| *AtSOS2* | ATTGAGGCTGTAGCGAAC | GGTATTCCTTCTGTTGCC | AT5G35410 |
| *AtSOS3* | GGAGGAATCTCTTCGCTG | CACGAAAGCCTTATCCACC | AT5G24270 |
| *AtFRY1* | CGCAGTAGCACTAGGATTG | TTGACACCGAGTTTATTGG | AT5G63980 |
| *AtADH* | CTCTTGGTGCTGTTGGTTTAGG | AATTGGCTTGTCATGGTCTTTC | AT1G77120 |
| *AtRD22* | GGTTCGGAAGAAGCGGAGAT | AGTGGAAACAGCCCTGACGT | AT5G25610 |
| *AtRD29A* | AAGCAATGAGCATGAGCAAG | GGAAGACACGACAGGAAACAC | AT5G52310 |
| *AtRD29B* | ACGACGGAAACATCGGACT | CTTCACCACCAGGAGCAAA | AT5G52300 |
| *AtERD1* | GTCAAGATGAGGCGGTAGC | GTCCACAGAAAAGCATAGCAG | AT5G51070 |
| *AtDREB2A* | AACCTGTCAGCAACAACAGC | AAACACATCGTCGCCATTTA | AT5G05410 |
| *AtNCED3* | TTGATGCTCCAGATTGCTTC | GGACCCTATCACGACGACTT | AT3G14440 |
| *AtPR-1* | AACTACGCTGCGAACACGTG | TCACTTTGGCACATCCGAGTC | AT2G14610 |
| *AtPdf1.2* | GAAGCACAGAAGTTGTGCGA | TGTAACAACAACGGGAAAATAAACA | AT5G44420 |
| *AtNPR1* | ACATCACCGGGTGTAAAGAT | AAGCCAGTTGAGTCAAGTCC | At1G64280 |
| *AtICS1* | CTTCCGTGACCTTGATCCTTTCT | CAGCGATCTTGCCATTAGGATC | AT1G74710 |
| *AtActin1* | GTCTGGATTGGAGGGTC | TGAGAAATGGTCGGAAA | AT2G37620 |
